# Economic burden of seasonal influenza B in France during winter 2010-2011

**DOI:** 10.1186/1471-2458-14-56

**Published:** 2014-01-20

**Authors:** Maria Laura Silva, Lionel Perrier, Hans-Martin Späth, Isidore Grog, Anne Mosnier, Nathalie Havet, Jean Marie Cohen

**Affiliations:** 1 GATE-UMR CNRS 5824, University of Lyon, University Lumière Lyon 2, Lyon 1, Lyon, France; 2 OPEN ROME, Coordination Nationale des Groupes Régionaux d’Observation de la Grippe, Réseau des GROG, Paris, France; 3 Cancer and Environment Department, Cancer Centre Léon Bérard, Lyon, France; 4 University of Lyon; University Claude Bernard Lyon 1, Lyon EAM 4128, France; 5 Collective name of members of GROG network, Paris, France

**Keywords:** Medical economics, Cost of illness, Influenza B, France, Health insurance reimbursement

## Abstract

**Background:**

In France, 2–15% of the population is affected annually by influenza, which causes significant socioeconomic disruption. Nevertheless, despite its importance for policy makers, few published studies have evaluated the impact of influenza B. Therefore, we assessed the costs associated with influenza B during 2010–2011 in France.

**Methods:**

Cases of lab-confirmed influenza B were analyzed as part of the Influenza B in General Practice Study. Cost calculations were based on micro-costing methods according to the French Health Insurance (FHI) perspective (in Euros, 2011). Costs were compared between age groups using the Kruskal–Wallis test, and when significant, by multiple comparisons based on rank. Moreover, uncertainties were assessed using one-way sensitivity and probabilistic analyses. Overall economic burden was estimated by multiplying cost per patient, flu attack rate, and the French population.

**Results:**

A total of 201 patients were included in the study. We found that the mean cost associated with Influenza B was 72€ (SD: 205) per patient: 70€ (SD: 262) for younger children, 50€ (SD: 195) for older children, 126€ (SD: 180) for adults, and 42€ (SD: 18) for elderly. Thus, we observed significantly different costs between the distinct age groups (p<0.0001). Finally, the economic burden of influenza B for the FHI was estimated to be 145 million Euros (95% CI: 88–201).

**Conclusions:**

Our findings highlight the important impact of influenza B and encourage further investigation on policy regarding vaccination strategies in France.

## Background

During seasonal influenza epidemics, it is estimated that 5–15% of the world population is affected by acute respiratory infections (ARIs) [1]. According to the World Health Organization (WHO) these annual epidemics result in 3–5 million cases of severe illness and 250–500 thousand deaths worldwide [1,2]. In France, during the 2010–2011 influenza season, the incidence of primary care medical consultations for lab confirmed flu was estimated to be 6.7% (6710/100,000; 95% CI: 4411–9009). This figure represented a typical attack rate in a medium intensity flu season when compared with data from recent years, which fluctuated between 2.5% (2007–2008) and 15% (2012–2013) [3,4].

Influenza viruses circulate during winter months (November–February in Europe), and epidemics last 8 weeks (on average) [4,5]. Generally, one circulating viral strain type or subtype is dominant during each season in a given location. However, in some seasons, there may be two or three different dominant viruses [6].

Patients with ARI induced by influenza, have the potential to develop a variety of complications, ranging from minor to life threatening [7]. Nevertheless, the majority of infected individuals experience only slight illness, which lasts less than two weeks and requires no medical intervention. In contrast, others may need medical consultations and/or work leave, but ultimately develop no complications [7]. However, influenza can be particularly serious in young children, the elderly, and people with chronic diseases. Indeed, these individuals can display an increased risk of severe complications, including pneumonia and fatal illness [8]. Due to the widespread affects of influenza, the national costs associated with the illness are of vital importance, especially when considering sickness benefit payments due to work leave and hospital/emergency care [8].

Policy makers are typically interested in the socioeconomic impact of influenza in order to set priorities for interventions [9]. However, few studies have evaluated the costs associated with illness and influenza vaccination programs [10,11]. Additionally, previous studies were rarely specific to particular viral strains [12,13]. In fact, few studies have provided strict investigations of influenza B or comparisons between influenza B and other viruses [14]. Furthermore, there have been recent discussions about whether or not to include two influenza B lineages within the seasonal influenza vaccine [15,16]. Therefore, to bridge the gap in knowledge regarding influenza B, a study entitled “Influenza B in General Practice” (IBGP) was initiated during the influenza season of 2010–2011. Notably, the study was designed to cover France and Turkey in its first phase. The overall aim of the IBGP was to analyze the morbidity and differential burden of illness (i.e., influenza-like illness consultations, prescriptions, sick leave) due to influenza B, including differences in age groups and lineages.

In this paper, we used data collected from the IBGP study to assess the impact of influenza illness on the French economy. Despite the fact that flu B represents 11% (median rate) of the detected influenza cases over the past eight years, no previous study has assessed the costs associated with lab-confirmed influenza B in France [4]. Although some studies regarding influenza B have been conducted in other countries [17,18], they are very specific to local practices and health systems, potentially hindering their relevance to the French system. Indeed, universal healthcare in France is largely financed by the National Health Insurance [19,20]. Therefore, our objective was to describe the costs associated with seasonal influenza B during the 2010–2011 season for a population presenting with ARI, consulting in primary care, under the perspective of the French Health Insurance (*Assurance Maladie*).

## Methods

### Study design

IBGP was an observational, prospective study conducted within sentinel surveillance networks in Europe. In France, the study was approved by the National Ethics Committee (N°911011) and the National Committee for Protection of Personal Data (N°11.016). Moreover, the study was proposed to all practitioners participating in the GROG network (*Groupes Régionaux d’Observation de la Grippe*), which included 390 general practitioners (GPs) and 116 pediatricians (2010–2011 season). These physicians were well distributed throughout the country and were trained to collect swab specimens and clinical information. Swabbed patients represented individuals with an ARI, defined as individuals consulting the practitioner within seven days (preferably two days) of a sudden onset of symptoms, including at least one systemic symptom (e.g., fever, headache, or myalgia) and one respiratory symptom (e.g., cough, rhinitis, or sore throat). As part of the routine surveillance for influenza during the whole season, swabbed patients were ultimately selected based on ad-hoc sampling, whereas systematic random sampling was used to select those patients to be swabbed. In this regard, each practitioner was required to swab the first ARI patient of each week within his/her specific age group: 0–4 years (GPs and pediatricians), 5–14 years (GPs and pediatricians), 15–64 years (GPs) and 65 years or more (GPs) [21].

The swabs and specimen request forms were then sent to the collaborating National Influenza Center (NIC, North and South) [21]. Virological methods were used for influenza identification and lineage characterization following the WHO Collaborating Centers recommendations [22]. The laboratories entered the clinical data and swab identification results into an electronic database. An anonymized version of this database was sent to GROG coordination [21]. Thus, this routine swab monitoring technique was used to identify influenza B cases and recruit patients for follow-up.

### Patients recruitment for follow-up

Following confirmation of an influenza B case, the study coordinator alerted the physician, who then made contact with the patient within 7 ± 2 days after the initial consultation. The patient was invited to participate in a follow-up assessment. For adults, oral consent was acceptable, whereas children required written parental agreement. For those patients who were not recovered at first follow-up contact, a further interview was arranged three weeks later. Thus, three study documents were used: the initial specimen request form (day 0; D0), the initial follow-up form (day 7 ± 2; D7), and the final follow-up form (day 28 ± 5; D28).

The D0 form included patient demographics, presence of similar cases in the household, vaccination status, presence of risk factors, and clinical symptoms [21]. The D7 and D28 follow-up forms were identical and were used to collect information regarding employment, ARI-related medical consults (e.g., telephone, medical office, or home visits), use of emergency services, hospitalization, additional tests, paramedical care, sick leave (work or school), drugs taken, duration of illness, and continuing symptoms [23].

### Cost assessment

The overall use of resources was determined based on data from three study documents (D0, D7, D28). Illness duration was defined from the date of illness onset to the recovery date, and costs were assessed from the French Health Insurance (FHI) perspective. Cost calculations (in Euros) were based strictly on a micro-costing approach [24], which involved analysis of FHI reimbursed fees [25] and the GROG methodology [23] (Box 1, Additional file 1). The following items were considered:

• Initial office consultation for ARI (GPs or pediatricians) [26];

• Follow-up office consultations [26];

• Home visits and additional consultations at the patient’s domicile [26];

• Telephone consultations (not cost allocated) [26];

• Emergency services (standard non-specific emergency care pack basis) [27];

• Hospitalizations (hospital care due to influenza complications) [28,29];

• Vaccine (cost of influenza vaccine, excluding administration) [30,31];

• Drugs taken (name and number of packs, assessed in three classes: antibiotics, antivirals, others [GROG methodology]). The lowest cost for each drug was used (age adjusted) [23,24,30,31];

• Additional tests costs (out of hospital) [32,33];

• Paramedical care costs (out of hospital) [26];

• Daily allowances (sick leave calculated per day after the fourth day of absence). The FHI calculates daily allowances based on patient’s gross wage, and when sickness leaves are > 31 days, on the number of dependent children. Conditions vary depending on the number of hours previously worked (< or > 200 hours during the previous three months) and the duration of sickness leave (< or > 6 months). However, in the present study the number of dependent children was not considered because sickness leaves were < 31 days. Also, we made the assumption that patients worked > 200 hours during the three previous months (this variable was not recorded) and obtained a sickness leave period lower than six months (i.e., flu sick leaves did not extend six months). Within this framework, the daily allowance was equal to 50% of the daily wage. When gross monthly earnings exceeded the maximum paid by the FHI, patient’s daily allowances were limited to the indemnity cap [34], which corresponded to 50% of the French mean daily wage (2830€ per month in 2011) [35].

The costs associated of influenza B per patient (the sum of reimbursements for each item) are summarized for four age groups: younger children (0–4 years), children (5–14 years), adults (15–64 years), and elderly (65 years or more).

A cost estimate for the whole French population [35] was obtained by applying this cost-related information to the national incidence estimate for influenza B as calculated by GROG [3,4,23] (Table 1).

**Table 1:** Estimation by age group of the incidence of primary care medical consultations for lab confirmed influenza B (per 100,000 inhabitants) extrapolated for the whole French population (winter 2010–2011)

	***Estimation of the incidence of consultations for influenza B***	***French population***	***Estimation of influenza B patients in French population***
	***[CI 95%]***		
Younger children	6.6%	3 884 625	255 834
(0–4 yo)	[4.3 – 8.9%]		[166 534 – 345 149]
Older children	12.9%	7 733 990	993 998
(5–14 yo)	[10.6 – 15.2%]		[816 477 – 1 172 086]
Adults	1.5%	40 808 626	612 246
(15–64 yo)	[0.8 – 3.8%]		[326 061 – 1 550 320]
Elderly	1.5%	10 661 749	157 268
(≥65 yo)	[0.8 – 3.8%]		[87 853 – 402 374]
All ages	3.2%	63 088 990	2 019 346
	[0.9 – 5.5%]		[552 660 – 3 453 491]

Source: GROG[3,4,23], INSEE [35]; yo = years old.

### Statistical analysis

Resource consumption and costs were summarized using descriptive statistics. Costs were compared between age groups using the Kruskal–Wallis test. Significant results were analyzed via multiple comparisons based on the rank. A significance value of 5% was retained, and one-way sensitivity analyses were conducted. Moreover, a variation of 20% was retained for each parameter value and illustrated graphically using a tornado diagram. The uncertainties surrounding the mean costs were assessed through probabilistic analyses using a non-parametric bootstrap method. A total of 1,000 simulated bootstrap samples were generated, and 95% confidence intervals were computed.

## Results

### Study population

#### Patient inclusion

During the 2010–2011 flu season in France we observed an outbreak of flu B/Victoria and A(H1N1)pdm09 viruses. These outbreaks peaked between week 51/2010 and week 08/2011. Overall, 153 sentinels participated in the study, recruiting patients between week 02/2011 and week 15/2011. A total of 460 swabs were confirmed as influenza B positive. Among these positive cases, 201 (44%) patients were successfully recruited: 115 were symptom free at D7, and 85 of the remaining 86 were followed at D28.

**Figure 1:**
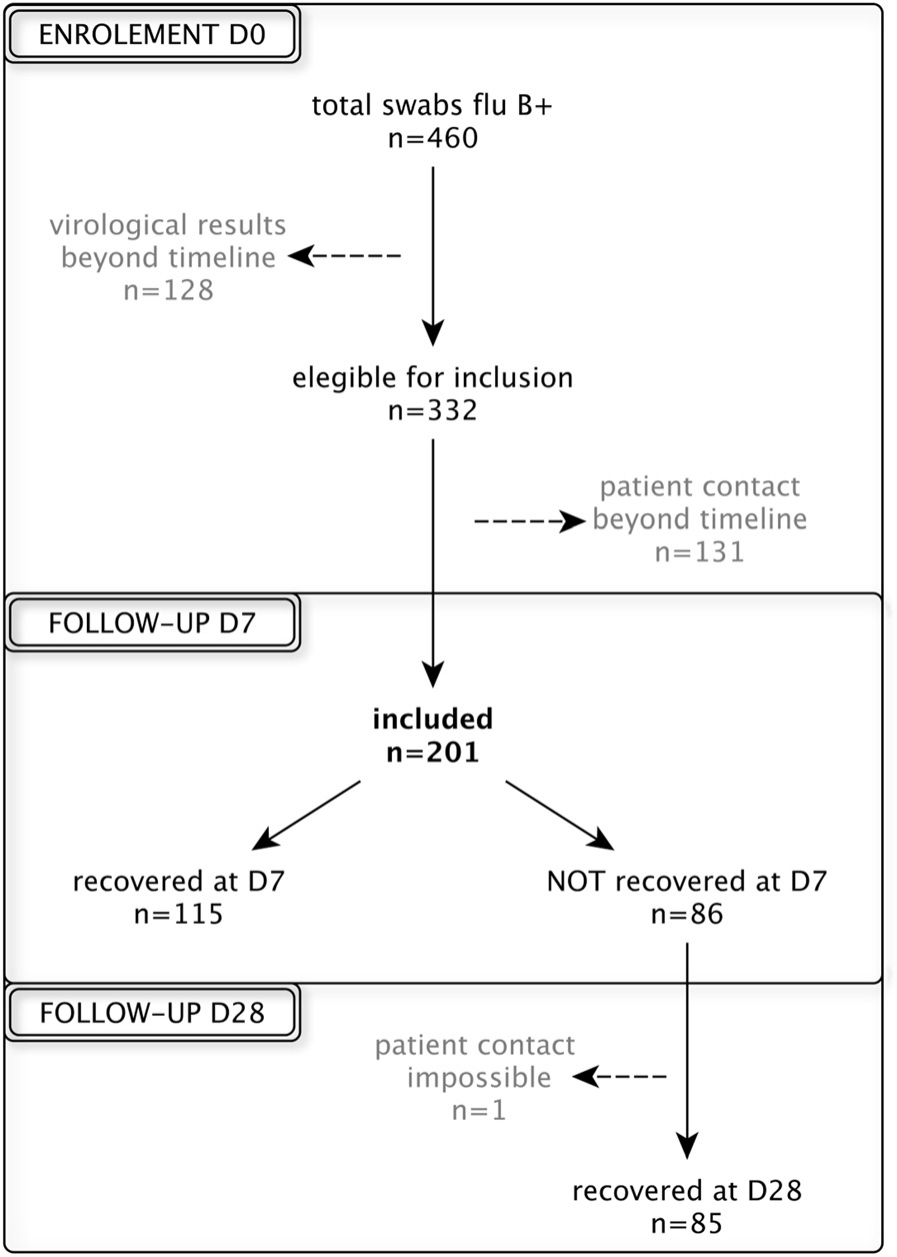
Flowchart for subjects included from D0 through the end of the follow-up period.

Furthermore, 56% of the lab-confirmed flu B swabs were not included (259), mainly due to the fact that the virology results were not available on time (nine days after swab) for the GROG coordination to invite physicians to recruit the patients (Figure 1).

#### Patients characteristics

The mean age of recruits was 17 years (extremes: 0–84). Seventy percent were children aged <15, 7% were elderly aged ≥ 65, and 23% were adults of working age, of whom 31 (over half) were employed with remuneration. Seven percent of the recruits were vaccinated against seasonal influenza, and 8% had a co-morbidity risk condition. Also, the M/F sex ratio was 1.25. Moreover, among the 20 women aged between 15 and 50, 3 were pregnant. A total of 115 patients (57%) recovered before day 9 (D9). The recovery rate before D9 was proportional: 63% of children, 43% of adults, and 13% of elderly (Table 2).

**Table 2:** Characteristics of patients included in the study

**Number of patients per age group and percentage of the total number**	***Younger children***		***Older children***		***Adults***		***Elderly***		***All ages***	
	***0-4 yo***		***5-14 yo***		***15*–*64 yo***		***≥65 yo***			
	***n***	***%***	***n***	***%***	***n***	***%***	***n***	***%***	***n***	***%***
	**50**	**25%**	**91**	**45%**	**46**	**23%**	**14**	**7%**	**201**	**100%**
Age (yo) mean ± SD	2.6 ± 1.3		9.3 ± 2.8		31.5 ± 13.7		72.1 ± 5.7		17.1 ± 19.7	
Risk factors	2	4%	5	5%	5	11%	5	36%	17	8%
*Pregnancy*	*NA*		*NA*		*3*		*NA*		*3*	
*BMI > 30*	*0*		*1*		*0*		*0*		*1*	
*Chronic disease*	*2*		*4*		*2*		*5*		*13*	
Working age (>15 y)	-	-	-	-	40	87%	14	100%	54	27%
*Employed*	-	-	-	-	31	78%	0	-	31	57%
Recovering at day 7 ± 2	37	74%	52	57%	20	43%	6	*43%*	115	57%

NA = non applicable; yo = years old; BMI = body mass index.

Almost 20% of patients reconsulted their family physician, with children requiring these additional consultations less than adults and the elderly. Patients mostly returned to their medical office or made telephone consultations. Very few emergency services (n = 3) and hospitalizations (n = 2) were observed, and only in children.

Drugs were taken by > 90% of patients, mainly for symptomatic relief. Overall, antibiotics were prescribed for 19% of the patients (all ages); but for the elderly 50%. Also, antivirals were prescribed in 20% of the cases.

Work leave was reported by 27 of 31 employed patients. These patients were out of work an average of 6.5 days (Box 2, Additional file 2).

#### Costs of influenza B

The estimated average cost of influenza B based on the FHI perspective was found to be 71.8€ (extremes: 16.1€ - 1876.6€) per patient. However, there were variations according to the distinct age groups (Table 3). In fact, costs were significantly different between age groups (p<0.0001). Costs related to antibiotic prescriptions were higher for those patients aged ≥ 65 years compared with the other age classes (p = 0.02).

**Table 3:** Mean costs per influenza B case per age group under the perspective of the French Health Insurance (in Euros, 2011)

	***0-4 yo (n = 50)***	***5-14 yo (n = 91)***	***15-64 yo (n = 46)***	***≥65 yo (n = 14)***	***All ages (n = 201)***	***p-value***
	***Mean (SD)***	***Mean (SD)***	***Mean (SD)***	***Mean (SD)***	***Mean (SD)***	
Direct costs (total)	70.0 (261.6)	50.0 (194.8)	33.4 (19.8)	41.8 (18.2)	50.6 (184.7)	<0.0003
*Initial consultation at medical office (GP or pediatrician)*	20.7 (1.7)	17.1 (1.6)	15.5 (1.0)	15.1 (0)	17.5 (2.4)	<0.0001
*Vaccine*	0.3 (1.3)	0.2 (1.1)	0.3 (1.3)	3.6 (3.2)	0.5 (1.7)	<0.0001
*Follow-up consultations at medical office (GP)*	3.3 (9.0)	3.5 (9.8)	7.0 (11.0)	11.9 (13.5)	4.9 (10.4)	0.0039
*Follow-up at domicile (GP)*	0.5 (3.6)	0.5 (4.8)	1.9 (7.8)	3.2 (11.8)	1.0 (6.1)	0.2718
*Emergency services*	0.0 (0)	1.2 (6.5)	0.0 (0)	0.0 (0)	0.5 (4.4)	0.3126
*Hospitalization*	36.0 (254.3)	19.8 (188.5)	0.0 (0)	0.0 (0)	17.9 (179.0)	0.7529
*Drugs (total)*	6.5 (10.3)	5.5 (8.8)	5.9 (5.5)	6.0 (6.2)	5.9 (8.4)	0.7705
*Antibiotics*	0.8 (2.2)	0.6 (1.8)	0.8 (2.0)	1.8 (2.9)	0.8 (2.0)	0.0214
* Antivirals*	0.7 (1.5)	0.8 (1.8)	2.3 (3.5)	1.1 (2.7)	1.1 (2.4)	0.0523
* Other drugs*	5.0 (9.2)	4.1 (8.5)	2.8 (3.3)	3.1 (3.9)	4.0 (7.6)	0.7518
*Additional tests*	2.8 (11.4)	2.2 (12.4)	2.8 (11.2)	2.1 (5.3)	2.5 (11.4)	0.5985
Daily allowances	0.0 (0)	0.0 (0)	92.7 (174.2)	0.0 (0)	21.2 (91.4)	<0.0001
*TOTAL*	*70.0(261.6)*	*50.0(194.8)*	*126.1(179.6)*	*41.8(18.2)*	*71.8 (205.1)*	*<0.0001*

yo = years old; GP = general practitioner.

Table 4 presents the mean drug-related costs for prescriptions containing at least one antibiotic. We observed that these costs were driven by antibiotic use in the entire population, with one antibiotic prescribed 46% of the time. However, when each age group was analyzed separately, antibiotics were also found to be cost drivers for children 0–4 years (47%) and adults (54%), but not for the remaining groups.

**Table 4:** Mean drug-related costs for prescriptions containing at least one antibiotic prescribed per influenza B case per age group under the perspective of the French Health Insurance (in Euros, 2011)

	***0-4 yo***		***5-14 yo***		***15-64 yo***		***≥65 yo***		***All ages***	
	***(n = 9)***		***(n = 16)***		***(n = 10)***		***(n = 3)***		***(n = 38)***	
	***Mean***	***%***	***€***	***%***	***€***	***%***	***€***	***%***	***€***	***%***
*Drugs (total)*	10.5		9.7		8.1		6.7		9.2	
*Antibiotics*	5.0	47%	4.3	44%	4.4	54%	1.2	19%	4.2	46%
*Antivirals*	2.5	24%	0.2	2%	1.5	19%	3.8	56%	1.4	15%
*Others*	3.0	29%	5.2	54%	2.2	27%	1.7	25%	3.6	39%

**Figure 2:**
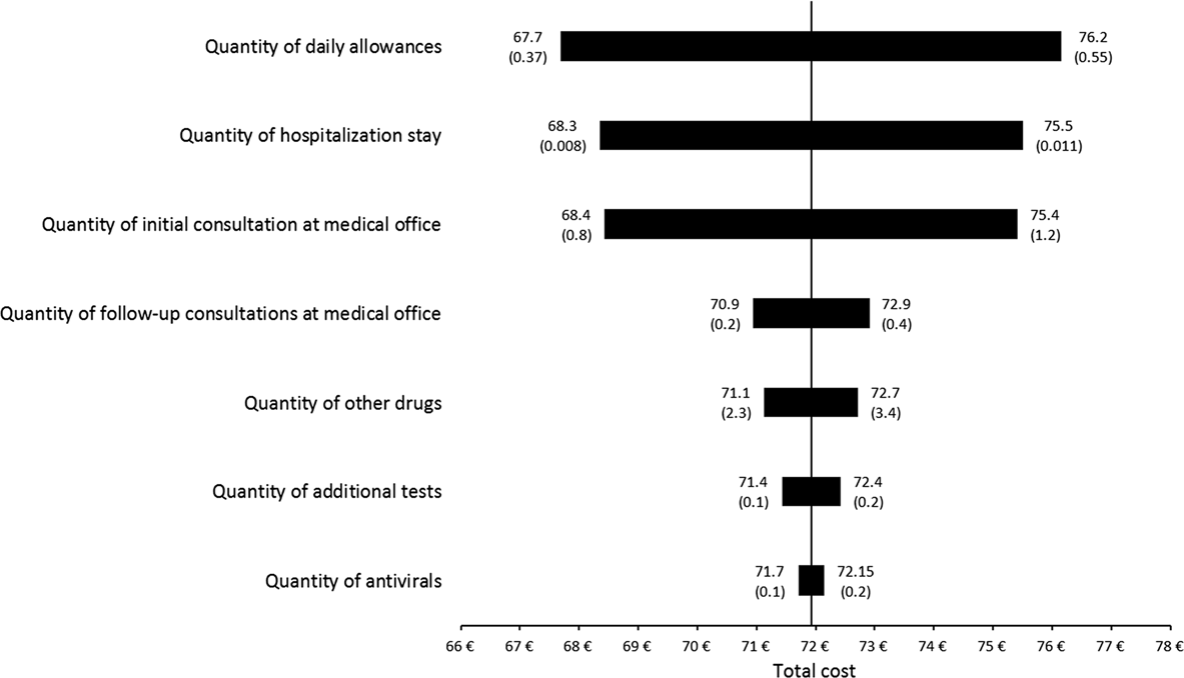
**Sensitivity of the cost of influenza B in France (tornado diagrams are used to graphically illustrate the impact of ±20% variation of the value of each parameter).** Legend: The length of the bar for each parameter represents the extent to which the mean of overall cost is sensitive to that particular variable. The graph presented so that the most influential parameter (the one with the longest bar) is on top. The vertical line represents the mean overall cost when all the parameters are at their base value.

In the tornado diagram (Figure 2), the vertical lines represent the mean cost when all parameters are fixed at their base value of 71.8€. Using this analysis, we observed that the most sensitive parameter was the quantity of daily allowances, which was followed by hospitalizations. In fact, increasing the amount of daily allowances by 20% increased the mean cost from 71.8€ to 76.2€. Using a non-parametric bootstrap method, the 95% confidence interval related to influenza B mean costs was found to be 43.6€ – 100.0€.

#### Extrapolation to French population

An extrapolation to the national population was based on an incidence rate of 3.2% of French people (2 million persons) with influenza B consulting a physician [4]. An estimation of the costs associated with influenza B per person (by age group and for all ages) is presented in Table 5. Notably, we estimated that the overall cost of influenza B to the FHI in 2010–2011 was 145 million Euros (95% CI: 88–201 millions €).

**Table 5:** Estimation of the cost of influenza B per age group under the perspective of the French Health Insurance (in Euros, 2011)

	**Population fluB + (IBGP)**	**Cost per patient (€) [CI 95%]**	**French population flu B + (estimation)**	Costs for the FHI* (€) [CI 95%]
*Younger children *	50	70.0	255 834	17 908 380
(0–4 yo)		[0.4 – 139.6]		[102 333 – 35 714 426]
*Older children*	91	50.0	993 998	49 699 900
*(5–14 yo)*		[12.0 – 88.0]		[11 927 976 – 87 471 824]
*Adults*	46	126.1	612 246	77 204 220
*(15–64 yo)*		[74.9 – 177.3]		[45 857 225 – 108 551 215]
*Elderly*	14	41.8	157 268	6 573 802
*(≥65 yo)*		[32.9 – 50.7]		[5 174 117 – 7 973 488]
*All ages*	201	71.8	2 019 346	144 989 042
		[43.6 – 100.0]		[88 043 485 – 201 934 600]

*costs include direct costs plus daily allowances for adults (more than 3 days of sick leave) under the perspective of the French Health Insurance (FHI); yo = years old.

## Discussion

Our study has estimated that the total impact of influenza B in France during the 2010–2011 winter season was 145 million Euros based on the FHI perspective. This finding demonstrates the important economic burden associated with influenza B in France. We observed that the cost generated by infected children exceeds that of other age groups when only direct costs are considered. Indeed, although only two children were hospitalized, hospitalizations were found to have the highest impact on costs. On the other hand, in adults, costs were mainly affected by daily allowances due to work leave.

### Limitations

Studies based on routine operational data can be exposed to potential biases, which might arise from the behavior of recruited subjects and/or selection by physicians. Recruitment to the study was initiated with a positive swab for influenza B. Thus, the extent to which the swabbing procedure might be selective is critical. Diagnoses were clinically based, and although guidance was given, strictly standard definitions are difficult due to the general symptoms of influenza.

Swab specimens were collected from consenting patients on an opportunistic basis. Therefore, these samples could have been influenced by several factors (e.g., the pressures of work and the time of the day). However, we are not aware of any bias that might have systematically prejudiced the incidence of laboratory-confirmed influenza positive samples. Moreover, GROG reports showed that ad-hoc and random selection of patients indicated good distribution based on age group [4].

Furthermore, limitations of the present study included possible selection bias for more susceptible patients (e.g., young infants and pregnant women) and reduced recruitments based on delays in the following processes: 1) transport of samples to the laboratory, 2) virological lab analysis, 3) transfer of lab results to the GROG, and 4) informing physicians of positive results (subsequently slowing patient contact).

Also, we have no further information, after the swab consultation, regarding the evolution of patients not included (259) in the study (e.g., if they had complications, if they were hospitalized). However, information collected (D0 form) on the swabbing day from patients not included in the study indicates that, in general, their characteristics were similar to our study population (Box 3, Additional file 3). Although the distribution of patients among age groups between the two populations was comparable, the proportion of adults was higher in the population not included. Moreover, the proportion of elderly was lower, but not significant. In addition, the proportion of males in the population not included was lower, with an insignificant M/F sex ratio of 1.02. There was also a significantly higher mean age for children (aged 5–14 years) included in the study (p < 0.001). Based on analysis of the population not included, it appears that no important differences in cost calculations or drivers were generated, except for perhaps the amount of overall costs.

Notably, our study only considered those patients consulting GROG physicians in general practice and pediatrics. Thus, patients directly admitted to hospitals or emergency departments (EDs) were not taken into account. According to the French Institute for Public Health Surveillance (InVS), during the influenza season studied, there were 17 019 ED visits for influenza-like illness and 919 hospitalizations observed within those services participating in the surveillance network. Overall, this represents 0.03% and 0.001% of the French population, respectively. Therefore, the weekly average proportion of hospitalizations for influenza-like illness among all hospitalizations was 0.14% [36]. In the present study, we found that the proportion of hospitalizations following an ARI consultation in primary care was 0.3%. Based on this data, the direct access of patients to EDs and hospitals due to influenza is not very important when considering care following primary consultation. Therefore, although the exclusion of those expenses represented a possible limitation of our study, it was not likely have a critical influence on our final results.

According to the National Council of the College of Physicians, there were 96 669 active physicians (GPs and pediatricians) working regularly in France during the time of this investigation [37]. Members of the GROG network for the 2010–2011 winter season included 506 volunteer GPs and pediatricians, and 30% of these participated in our study. The two referenced laboratories partners of the GROG network provided 60% of the virological information [4]. Although our entire study population was made up of patients with lab-confirmed influenza B, they came from specific regions of France, representing 65% of the country (NIC North and South).

### Patient characteristics, healthcare consumption and sick leave

A total of 6.7% of the French population was affected by influenza during our study period, and two influenza viruses were dominant: influenza B (3.2%) and influenza A(H1N1)pdm09 (3.5%). Notably, our investigation corresponded to a post-pandemic influenza season, which may have influenced the attitudes of patients and physicians (e.g., higher rate of antiviral prescriptions) [4]. Nevertheless, according to the GROG network, the number of consultations due to ARI was not higher than the usual rate observed during ordinary annual influenza outbreaks [4]. However, children were more likely to consult physicians than adults, which may have biased the average age of our study population. Consequently this may have influenced our results regarding higher costs for children.

Also, the small sample of elderly patients included may have resulted from a high vaccination coverage (54% in France 2011), which could have decreased influenza complications and consultations in this age group. In addition, elderly usually consult later than 48 h following the onset of symptoms. Indeed, this factor represents an important exclusion criterion for specimen collection in the GROG network [38].

Although the rate of employment in our working age population (67%) was rather high when compared to the French employment rate (58%), it was not statistically significant [35]. Notably, a lower employment rate (54%) was observed during a GROG study conducted during the 2005–2006 influenza season (also influenza B as dominant virus) [3], but the difference was also found to be insignificant when compared to the general French employment rate. We believe that our sample was representative of the general population; however, our costs may have possibly been overestimated.

From a societal perspective, each case of influenza in working people leads to between three and seven lost working days [39]. In Europe, influenza accounts for approximately 10% of sickness-related absences from work, while the cost of productivity lost in France and Germany has been estimated to be in the range of 9.3–14.1 billion USD per year [39]. Our results are comparable with these previous findings: adults with remunerated jobs displayed an average of 6.5 days of sick leave, which represented 13% of the population absent from work. In addition, we also calculated the work days lost by parents as a result of child illness. Although the FHI provides no daily allowances in these cases, it is important to state that more than one in four parents (children < 14 years) were out of work for approximately 2.8 days under these circumstances. In fact, a recent socioeconomic study conducted in Hong Kong with hospitalized children reported an average of 5.3 ± 3.6 days of school leave for patients with lab-confirmed influenza B [40]. Indeed, our estimated school leave for treated children was similar (5.6 days).

The indemnity cap considered for daily allowances was critical for our obtained results. Since we were not allowed to collect personal information from the included patients, we considered French mean daily wage in 2011 [35]. Since the indemnity cap may not exceed the upper limit of daily allowances paid by the FHI, we applied the latter. We believe that careful considerations should be taken into account when extrapolating our results, due to possible over or underestimation of these cost calculations.

### Cost of influenza B

The cost drivers for each age group varied. Our findings revealed that hospitalization was the major driver (51%) in younger children, whereas for older children costs were driven by hospitalization (40%) and initial consultation (34%). Daily allowances represented 73% of costs in adults. In contrast, for the elderly, the main driver was medical consultations (72%). Some authors have found similar drivers; however, others have also identified costs associated with vaccine administration, which was not assessed in our study [41,42].

Levy reported the only similar economic analysis of the burden of influenza illness in France for the seasons between 1985 and 1989. In the study by Levy, costs were estimated exclusively based on clinical incidence data related to influenza-like illness from the perspectives of both FHI and society [19]. Indeed, the results of our study are comparable with those presented by Levy. Both studies highlight to the economic importance of sick leave: first three days paid by the employer, followed by the FHI after the fourth day. According to Levy, FHI carried 70% of costs associated with influenza and the remaining 30% referred to the societal perspective (FHI excluded). This suggests that if we had measured the societal perspective in our study, the costs of influenza B might have increased by 43%. Thus, a further analysis of all indirect costs, including lost productive capacity and costs associated with the employment of substitute workers, will be needed in future studies. Additionally, different perspectives (e.g., patient, employer, private and mutual funds) could be considered.

Furthermore, Carrat et al. published an influenza burden of illness study that intended to obtain data for improving the cost-effectiveness of strategies against the disease, but no cost analyses were performed. Nevertheless, in the same study, the authors found that the mean number of sick leave for working adults was 4.0 ± 2.8 days [20]. In comparison, in our study, we found a higher duration (6.5 days) of sick leave among working age patients.

According to the French Ministry of Health, during the 2010–2011 influenza season, nearly 10 million people were targeted by the national influenza vaccination program, receiving an invitation from the French Health Insurance to obtain free vaccination. Among them, 51.8% were vaccinated [43]. Considering this vaccination coverage (approximately 5 million people), we estimate that the expenses related to vaccine cost and administration for the FHI would be around 110 million Euros. This amount is likely to be underestimated, as we did not consider expenses related to the national influenza program, institutional campaigns, postal services, and other indirect costs. Indeed, there is no public information available related to theses expenses. However, according to the estimates described above, we suggest that investments on vaccination strategies are still less expensive than the costs avoided with influenza care.

## Conclusions

To our knowledge, this is the first cost study specifically related to influenza B in France. Our findings highlight avoidable costs related to influenza and are valuable in the context of evaluating healthcare interventions and public health strategies using economic models.

There is a lack of published literature about the costs associated with different viral strains of influenza. Thus, further knowledge is crucially required for policy makers to effectively decide on strategies regarding influenza (e.g., market access and reimbursement for new vaccines, implementation of vaccination programs in a pandemic situation). Therefore, we propose that refined studies targeting influenza economics should be developed in order to facilitate the work of policy-makers.

Furthermore, our results have the potential to influence decisions concerning seasonal influenza vaccine formulation. Currently, the most common available seasonal influenza vaccine contains only one lineage of influenza B (together with two flu A strains) [22]. Immunization against B virus strains of one lineage provides limited cross-protection against strains of the other lineage [18]. For this reason, and the difficulty of predicting which B virus lineage will predominate during a given season, vaccines containing two influenza B strains (together with two flu A strains) are recently receiving marketing authorization [15,18]. Therefore, policy-makers are evaluating the benefit of adopting those vaccines into their national influenza programs. Further investigations into the impact of such quadrivalent vaccines and vaccine effectiveness will be required.

## Competing interests

The authors declare that they have no competing interests.

## Authors’ contributions

JMC and AM designed the IBGP study. IG, MLS, and AM acquired and analyzed clinical data. MLS, LP, and AM participated in cost data acquisition. MLS, LP, HMS, AM, NH, and JMC performed the statistical analysis and prepared the manuscript. All authors read and approved the final manuscript.

## Pre-publication history

The pre-publication history for this paper can be accessed here:

http://www.biomedcentral.com/1471-2458/14/56/prepub

## Supplementary Material

Additional file 1: Box 1 The unit costs of analyzed items [26–35] Legend: ATU: Reception and treatment of emergencies; CCAM: Classification commune des actes médicaux; FHI: French Health Insurance; GHM: Groupes Homogènes de Malades; GHS: Groupes Homogènes de Séjour; GP: General Practice[tioner]; GROG: Groupes Régionaux d’Observation de la Grippe; ICD: International Common Denomination; MCCO: activities of medicine, surgery, obstetrics and dentistry; MGE: supplement for children 2–6 years; MNO: supplement for children 0–2 years; NGAP: Nomenclature Générale des Actes Professionnels; sector 1: corresponds to the rate that is the basis for the reimbursement of health insurance; TNB: Table National de Biologie; yo: years old. Click here for file

Additional file 2: Box 2 Healthcare consumption and sick leave per age group during the entire study period. Click here for file

Additional file 3: Box 3 Characteristics of patients presenting with ARI consulting a GROG practitioner but NOT included in the study (259) in comparison with patients included in the study (201). Click here for file
