# Supplementary material for: Economic burden of seasonal influenza B in France during winter 2010-2011
Source: BMC Public Health. 2014 Jan 20;14:56. doi: 10.1186/1471-2458-14-56 (PMC3909302; doi:10.1186/1471-2458-14-56)
Supplement: Additional file 1: Box 1 — The unit costs of analyzed items [26–35] Legend: ATU: Reception and treatment of emergencies; CCAM: Classification commune des actes médicaux; FHI: French Health Insurance; GHM: Groupes Homogènes de Malades; GHS: Groupes Homogènes de Séjour; GP: General Practice[tioner]; GROG: Groupes Régionaux d’Observation de la Grippe; ICD: International Common Denomination; MCCO: activities of medicine, surgery, obstetrics and dentistry; MGE: supplement for children 2–6 years; MNO: supplement for children 0–2 years; NGAP: Nomenclature Générale des Actes Professionnels; sector 1: corresponds to the rate that is the basis for the reimbursement of health insurance; TNB: Table National de Biologie; yo: years old. [file 1471-2458-14-56-S1.pdf]

Box 1: The unit costs of analyzed items [26-35]

Items	*Unit costs (€)*	*Reimbursement basis of the FHI (general cases)*	*Unit cost paid by the FHI (€)*	*Reference source*	*Comments*
**Initial consultation by GP**				*Assurance Maladie – NGAP* [26]	Considering the family doctor of the patient attending at his office [sector 1]
<2 yo	28.00	70%	19.60		
2-6 yo	26.00	70%	18.20		
6-18 yo	23.00	70%	16.10		
>18 yo	23.00	70% [23.00€ * 70%= 16.10€ – 1.00€]	15.10		Patients over 18 yo have a fee of 1€ charged
**Initial consultation by PEDIATRICIAN**				*Assurance Maladie* [26]	Considering a physician attending at his office [sector 1]
<2 yo	31.00	70% and 100% [26.00€ * 70%+ 5.00€ * 100%]	23.20		Children under 2 yo have pediatric child fees of 5€ 100% reimbursed
2-6 yo	31.00	70%	21.70		
6-16 yo	28.00	70%	19.60		
**Vaccine**				*Vidal* [30] *Thériaque* [31]	Cost of the vaccine, but not the cost of administration (due to the complexity of measurement)
≥65 yo or risk morbidity	6.25	100%	6.25		
others	6.25	0%	0		
**Follow-up consultation**				*Assurance Maladie* [26]	Considering the family GP attending at sector 1
<2 yo	28.00	70%	19.60		
2-6 yo	26.00	70%	18.20		
6-18 yo	23.00	70%	16.10		
>18 yo	23.00	70% [23.00€ * 70%= 16.10€ – 1.00€]	15.10		Patients over 18 yo have a fee of 1€
**Telephone consultation**	-	0	0.00	*Assurance Maladie* [26]	Phone consultations are not charged

Items	*Unit costs (€)*	*Reimbursement basis of the FHI (general cases)*	*Unit cost paid by the FHI (€)*	*Reference source*	*Comments*
**Home visit**					
<2 yo	38.00	70%	26.60	*Assurance Maladie* [26]	visit [23€] + shifting [10€] + MNO [5€]
2-6 yo	36.00	70%	25.20		visit [23€] + shifting [10€] + MGE [3€]
6-18 yo	33.00	70%	23.10		visit [23€] + shifting [10€]
>18 yo	33.00	70% [33.00€ * 70%= 23.10€ – 1.00€]	22.10		visit [23€] + shifting [10€] - 1€ of consultation fees charged
**Emergency services**				*Ministère de la Santé* [27]	Package for attending non expected patients at a medical institution without following hospitalization [ATU at MCOO]
**general costs**	25.28	80%	20.22		
<2 yo / *consult + ER*	28.00+ 25.28	70% 80%	39.82		
2-6 yo / *consult + ER*	26.00+ 25.28	70% 80%	38.42		Considering usual consultations fees
6-18 yo / *consult + ER*	23.00+ 25.28	70% 80%	36.32		
>18 yo / *consult + ER*	23.00+ 25.28	70% - 1.00€ 80%	35.32		
**Hospitalization**	2248.16	80% [independent of number of days]	1798.53	*Assurance Maladie* [28] *Ministère de la Santé* [29]	Package for treating respiratory signs and symptoms without other morbidities [GHS 1116, GHM 04M11V]

Items	*Unit costs (€)*	*Reimbursement basis of the FHI (general cases)*	*Unit cost paid by the FHI (€)*	*Reference source*	*Comments*
**Drugs**	*Only reimbursed drugs were considered: reimbursement rate is applied for the cheapest ICD by age group*				
**ANTIBIOTICS** [example of **amoxiciline**]				*GROG* [21] *Vidal* [30] *Thériaque* [31]	-
<3 yo	1.60	65%	1.04		
3-6 yo	2.00	65%	1.30		
>6 yo	2.06	65%	1.34		
**ANTIVIRALS** [example of **oseltamivir** as curative treatment]				*GROG* [21] *Vidal* [30] *Thériaque* [31]	30% of reimbursement is considered for all patients
<12 yo	12.69	30%	3.81		
≥12 yo	24.85	30%	7.46		
**OTHERS** [example of **paracetamol**]				*GROG* [21] *Vidal* [30] *Thériaque* [31]	
<8 yo	1.93	65%	1.25		
≥8 yo	1.47	65%	0.96		
**Additional tests**					
Creatine Phosphokinase	3.24 + 4.05	60%	4.37	*Assurance maladie* [32] *TNB*	Code: CPK 1520 Subscription: 12B [12*0.27€] One exam for each patient + blood taken 15B [4.05€]
Cysteine-Rich Protein	5.40 + 4.05	60%	5.67		Code: CRP 1804 Subscription: 20B [20*0.27€] One exam for each patient + Blood taken 15B [4.05€]

Items	*Unit costs (€)*	*Reimbursement basis of the FHI (general cases)*	*Unit cost paid by the FHI (€)*	*Reference source*	*Comments*
Urine culture	18.90	60%	11.34		Code: ECBU 5201 Subscription: 70B [70*0.27€] One exam for each patient
Electrocardiogram	13.52 + 23.00	70%	25.56		Code: DEQP001 One exam for each patient + labor costs [23€]
Chest X Ray	25.92 + 23.00	70%	34.24	*Assurance maladie* [33] *CCAM*	Code: ZBQK002 One exam for each patient + labor costs [23€]
Cranial X Ray	23.94 + 23.00	70%	32.86		Code: LAQK003 One exam for each patient + labor costs [23€]
Thorax Scanner [without contrast intravenous injection]	30.78 + 23.00	70%	37.65		Code: ZBQK001 One exam for each patient + labor costs [23€]
**Paramedical care**					
Respiratory physiotherapy and physiotherapist fees	103.20	60%	75.72	*Assurance Maladie* [26] *NGAP*	Code: AMK 8 *average package of 6 sections* *2.15€ x 8 € x 6 sections* One pack for each patient
Nutritionist	-	0	0.00	-	Consultation to nutritionist is not reimbursed
Osteopathy	-	0	0.00	-	Consultation to osteopath is not reimbursed
**Work absenteeism**	-	-	46.33	*Assurance Maladie* [34] INSEE [35]	Cost per day for patients in remunerated employment Since the 4th day of absence

Legend: ATU: Reception and treatment of emergencies; CCAM: Classification commune des actes médicaux; FHI: French Health Insurance; GHM: Groupes Homogènes de Malades; GHS: Groupes Homogènes de Séjour; GP: General

Practice[tioner]; GROG: Groupes Régionaux d'Observation de la Grippe; ICD: International Common Denomination; MCCO: activities of medicine, surgery, obstetrics and dentistry; MGE: supplement for children 2-6 years; MNO: supplement for children 0-2 years; NGAP: Nomenclature Générale des Actes Professionnels; sector 1: corresponds to the rate that is the basis for the reimbursement of health insurance; TNB: Table National de Biologie; yo: years old.
