# Supplementary material for: Economic burden of seasonal influenza B in France during winter 2010-2011
Source: BMC Public Health. 2014 Jan 20;14:56. doi: 10.1186/1471-2458-14-56 (PMC3909302; doi:10.1186/1471-2458-14-56)
Supplement: Additional file 2: Box 2 — Healthcare consumption and sick leave per age group during the entire study period. [file 1471-2458-14-56-S2.pdf]

Box 2: Healthcare consumption and absenteeism per age group during the entire study period

| | *Younger children 0-4 yo (n=50)* | *Older children 5-14 yo (n=91)* | *Adults 15-64 yo (n=46)* | *Elderly ≥65 yo (n=14)* | *All ages (n=201)* |
|---|---|---|---|---|---|
| **Initial consultation at GP or Pediatrician** | | | | | |
| CONSULTATION AT THE MEDICAL OFFICE | | | | | |
| *number of patients (% of total patients)* | 50 (100%) | 91 (100%) | 46 (100%) | 14 (100%) | 201 (100%) |
| *number of contacts (total)* | 1 | 1 | 1 | 1 | 1 |
| **Vaccine** | | | | | |
| *number of patients (% of total patients)* | 2 (4%) | 3 (3%) | 2 (4%) | 8 (57%) | 15 (7%) |
| **Follow up contacts with physician investigator** | | | | | |
| CONSULTATION AT THE MEDICAL OFFICE | | | | | |
| *number of patients (% of total patients)* | 6 (12%) | 11 (12%) | 10 (22%) | 8 (57%) | 35 (17%) |
| *number of contacts (total)* | 8 | 17 | 15 | 11 | 50 |
| *mean ±SD* | 1.3±0.8 | 1.5±0.7 | 1.4±0.5 | 1.4±0.5 | 1.4±0.6 |
| *min-max* | 1-3 | 1-3 | 1-2 | 1-2 | 1-3 |
| CONSULTATION BY TELEPHONE | | | | | |
| *number of patients (% of total patients)* | 4 (8%) | 13 (14%) | 11 (2%) | 5 (36%) | 33 (16%) |
| *number of contacts (total)* | 7 | 19 | 16 | 6 | 48 |
| *mean ±SD* | 1.7±1.5 | 1.5±0.9 | 1.5±0.7 | 1.2±0.4 | 1.5±0.8 |
| *min-max* | 1-4 | 1-4 | 1-3 | 1-2 | 1-4 |
| CONSULTATION BY HOME VISIT | | | | | |
| *number of patients (% of total patients)* | 0 | 1 (1%) | 2 (4%) | 1 (7%) | 4 (2%) |
| *number of contacts (total)* | 0 | 2 | 3 | 2 | 7 |
| *mean ±SD* | - | - | 1.5±0.7 | - | 1.7±0.5 |
| *min-max* | - | - | 1-2 | - | 1-2 |
| **Follow up contacts with other physician** | | | | | |

| | *Younger children 0-4 yo (n=50)* | *Older children 5-14 yo (n=91)* | *Adults 15-64 yo (n=46)* | *Elderly ≥65 yo (n=14)* | *All ages (n=201)* |
|---|---|---|---|---|---|
| CONSULTATION AT THE MEDICAL OFFICE | | | | | |
| *number of patients (% of total patients)* | 0 | 4 (4%) | 3 (6%) | 1 (7%) | 8 (4%) |
| *number of contacts (total)* | 0 | 6 | 3 | 1 | 10 |
| *mean ±SD* | - | 1.5±0.6 | 1 | - | 1.3±0.4 |
| *min-max* | - | 1-2 | 1-1 | - | 1-2 |
| CONSULTATION BY HOME VISIT | | | | | |
| *number of patients (% of total patients)* | 1 (2%) | 0 | 1 (2%) | 0 | 2 (1%) |
| *number of contacts (total)* | 1 | 0 | 1 | 0 | 2 |
| *mean ±SD* | - | - | - | - | 1 |
| *min-max* | - | - | - | - | 1-1 |
| **Emergency services** | | | | | |
| *number of patients (% of total patients)* | 0 | 3 (3%) | 0 | 0 | 3 (2%) |
| **Hospitalization** | | | | | |
| *number of patients (% of total patients)* | 1 (2%) | 1 (1%) | 0 | 0 | 2 (1%) |
| **Drugs** | | | | | |
| TOTAL DRUGS TAKEN | | | | | |
| *number of patients (% of total patients)* | 45 (90%) | 86 (94%) | 42 (91%) | 13 (93%) | 186 (93%) |
| ANTIBIOTICS | | | | | |
| *number of patients (% of total patients)* | 8 (16%) | 14 (15%) | 9 (19%) | 7 (50%) | 38 (19%) |
| ANTIVIRALS | | | | | |
| *number of patients (% of total patients)* | 9 (18%) | 15 (16%) | 14 (30%) | 2 (14%) | 40 (20%) |
| OTHERS | | | | | |
| *number of patients (% of total patients)* | 45 (90%) | 84 (92%) | 42 (91%) | 13 (93%) | 184 (92%) |
| **Additional tests** | | | | | |
| *number of patients (% of total patients)* | 4 (8%) | 5 (5%) | 5 (11%) | 2 (14%) | 16 (8%) |
| **Paramedical care** | | | | | |
| *number of patients (% of total patients)* | 0 | 0 | 0 | 0 | 0 |

| | ***Younger children 0-4 yo (n=50)*** | ***Older children 5-14 yo (n=91)*** | ***Adults 15-64 yo (n=46)*** | ***Elderly ≥65 yo (n=14)*** | ***All ages (n=201)*** |
|---|---|---|---|---|---|
| **Sick leave (from work)** | | | | | |
| *number of patients (% of total patients)* | - | - | 27 (59%) | 0 | 27 (13%) |
| *average duration (days±SD)* | - | - | 6.5±5.4 | 0 | 6.5±5.4 |
| *min-max* | - | - | 1-24 | 0 | 1-24 |

yo: years old
