# Supplementary material for: Economic burden of seasonal influenza B in France during winter 2010-2011
Source: BMC Public Health. 2014 Jan 20;14:56. doi: 10.1186/1471-2458-14-56 (PMC3909302; doi:10.1186/1471-2458-14-56)
Supplement: Additional file 3: Box 3 — Characteristics of patients presenting with ARI consulting a GROG practitioner but NOT included in the study (259) in comparison with patients included in the study (201). [file 1471-2458-14-56-S3.pdf]

Box 3: Characteristics of patients presenting with ARI consulting a GROG practitioner but NOT included in the study (259) in comparison with patients included in the study (201)

	Younger children 0-4 yo			***Older children 5-14 yo***			***Adults 15-64 yo***			***Elderly ≥65 yo***			***All ages***		
	NOT included	***included***	***p***	***NOT included***	***included***	***p***	***NOT included***	***included***	***p***	***NOT included***	***included***	***p***	***NOT included***	***included***	***p***
**Nb of patients** (% per age group)	67 (26%)	50 (25%)	-	112 (44%)	91 (45%)	-	67 (26%)	46 (23%)	-	10 (4%)	14 (7%)	-	256* (100%)	201 (100%)	-
**Age (yo) mean±SD**	2.6±1.3	2.6±1.3	0.95	7.9±2.7	9.3±2.8	<0.001	33.6±14.8	31.5±13.7	0.40	68.4±3.8	72.1±5.7	<0.1	15.6±18.0	17.1±19.7	0.37
**Vaccine**** (number vaccinated)	3	2	-	4	3	-	4	2	-	2	8	-	13	15	0.15
**Risk Factors**** (number with risk)	1	2	-	5	5	-	7	5	-	1	5	-	14	17	<0.1
pregnancy	NA	NA	-	NA	NA	-	2	3	-	NA	NA	-	2	3	-
BMI>30	0	0	-	0	1	-	2	0	-	1	0	-	3	1	-
chronic disease	1	2	-	5	4	-	3	2	-	0	5	-	9	13	-

* Description is based on 256 patients (from 259 patients not included, 3 did not report date of birth)

**p value not calculated due to small sample size (<10)

yo: years old
